# Silent severe aortic regurgitation due to blunt chest trauma: ignore it at your peril—a case report

**DOI:** 10.1093/ehjcr/ytae499

**Published:** 2024-09-17

**Authors:** Rafaella I L Markides, Ulrich P Rosendahl, Isabelle Roussin

**Affiliations:** University of Cambridge School of Clinical Medicine, Box 111 Cambridge Biomedical Campus, Cambridge CB2 OSP, UK; Department of Cardiac Surgery, Royal Brompton Hospital, Guy’s and St Thomas’ NHS Foundation Trust, London SW3 6NP, UK; Department of Cardiology, Lister Hospital, East & North Hertfordshire NHS Trust, Coreys Mill Lane, Stevenage SG1 4AB, UK

**Keywords:** Blunt chest trauma (BCT), Aortic regurgitation (AR), Emergency echo, Valve trauma, Road traffic accident (RTA), Aortic valve, Case report, Surgical aortic valve replacement

## Abstract

**Background:**

Blunt chest trauma (BCT) presenting to the emergency department is common and may cause life-threatening cardiac complications. Whilst complications causing haemodynamic instability are generally detected promptly, others may present late with long-term consequences. We describe a rare, serious complication of BCT presenting five years after a road traffic accident (RTA).

**Case summary:**

A 23-year-old man was incidentally found to have a murmur. Past history was notable only for BCT with rib fracture sustained in a RTA 5 years prior. Examination revealed a hyperdynamic pulse, loud decrescendo diastolic murmur, and Duroziez’s sign over the femoral arteries. Echocardiography showed severe valvular aortic regurgitation (AR) from a hole in the left coronary cusp and holodiastolic flow reversal in the descending aorta. The left ventricle (LV) showed marked dilatation in diastole, mild dilatation in systole, and preserved systolic function. The aorta was normal. Severe AR was attributed to his previous BCT, with AR causing subsequent LV dilatation. He underwent aortic valve replacement (AVR) with rapid recovery. He remains well, and his echo shows a well-functioning AVR with normalization of LV dimensions.

**Discussion:**

Aortic regurgitation following BCT is rare but well-recognized, most often resulting from RTAs. Only a third of cases are diagnosed acutely. In others, lack of haemodynamic instability means that emergency echocardiography is not routinely performed, such that this may go unrecognized with long-term consequences. Clinicians should be aware of possible valve damage following BCT. Prompt echocardiography should be routinely performed for all BCT at initial presentation, even without haemodynamic instability.

**Learning points**

- Aortic valve trauma resulting in severe aortic regurgitation is a rare but well-recognized consequence of blunt chest trauma (BCT), most often resulting from road traffic accidents.
- Clinicians should be highly vigilant for possible valve damage following BCT, even in the absence of immediate haemodynamic instability, and prompt echocardiography should be performed in all cases.
- Surgical aortic valve replacement is the treatment of choice after multidisciplinary decision for severe aortic regurgitation.

## Introduction

Blunt chest trauma (BCT) is often seen in the emergency department (ED), and well-known to potentially result in cardiac injury.^1^ Acutely life-threatening cardiac complications generally give rise to haemodynamic instability, such as cardiac tamponade, and hence tend to be detected promptly. However, other serious cardiovascular complications may go undetected, present late, and potentially cause long-term consequences if undiagnosed.^2^ We present a rare, serious complication of BCT in a young individual presenting five years after a road traffic accident (RTA).

## Summary figure

**Figure:**
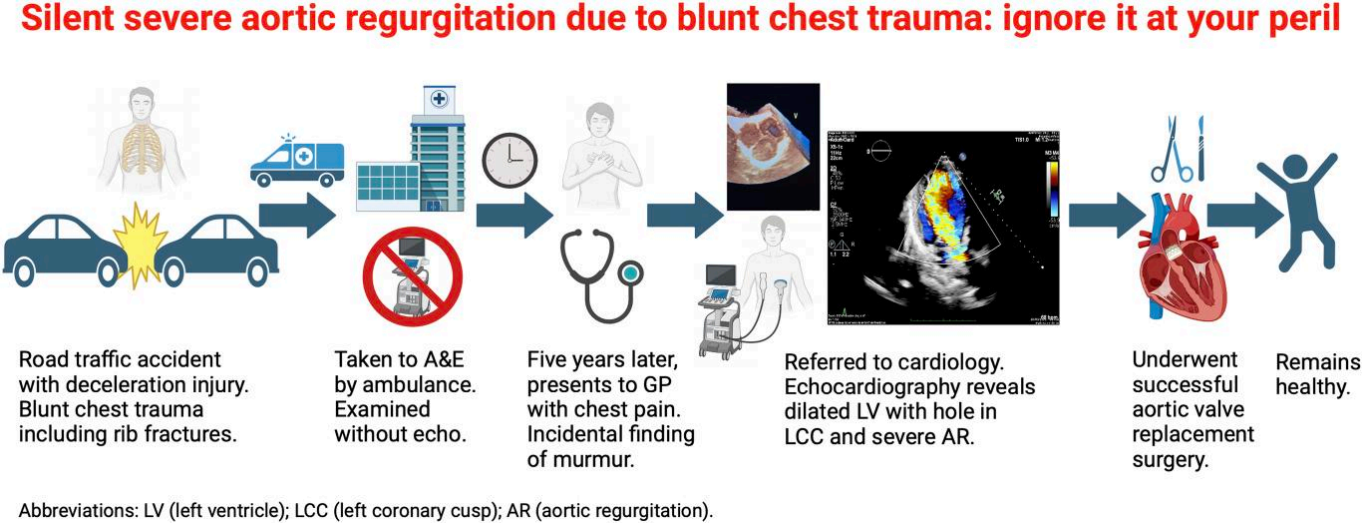


## Case presentation

This 23-year-old gentleman presented to his GP in March 2023 with pain over the left ribcage which he felt to be muscular in nature, as there was localized tenderness on palpation. The GP detected a heart murmur on auscultation and a displaced apex beat and referred him to cardiology. His past medical history was asthma and a RTA five years prior, in which he sustained BCT with rib fracture. There was no other past medical history, including no drug, allergy, or family history. He did not smoke, drink alcohol, or use recreational drugs. He was otherwise fit and well, undertaking manual labour and having recently run a half marathon without symptoms.

Notable findings on examination were a hyperdynamic pulse with a loud decrescendo diastolic murmur, loudest at the left sternal edge, and Duroziez’s sign over the femoral arteries.

**Figure 1:**
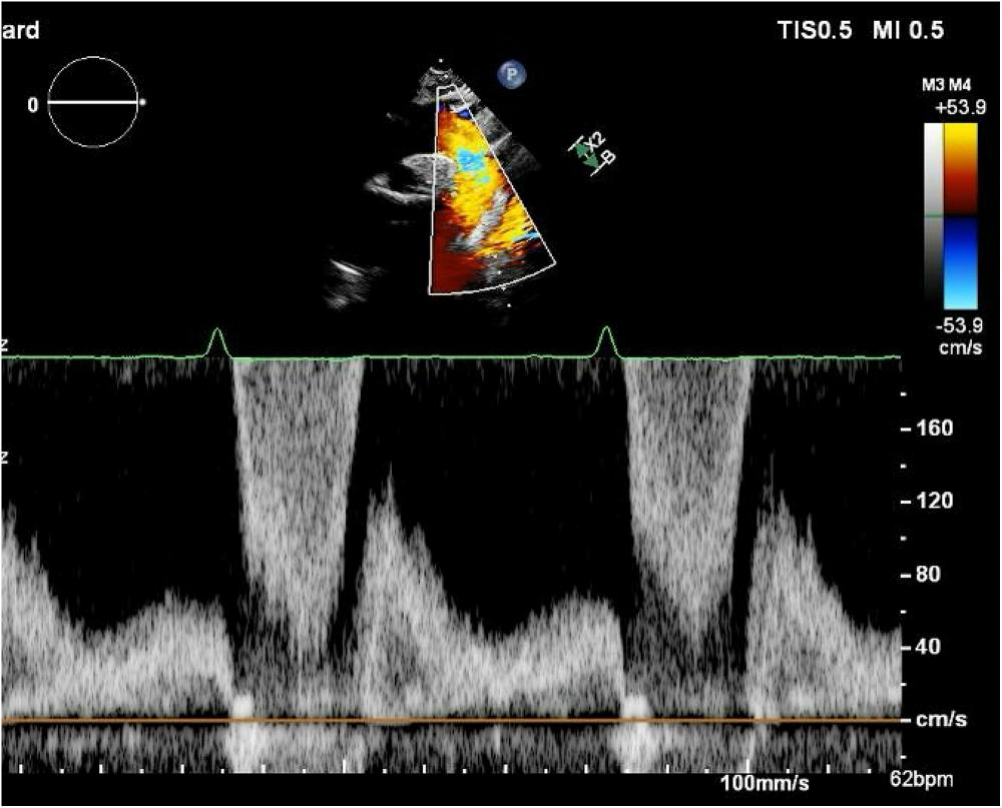
Doppler signal in aorta showing holodiastolic flow reversal (TTE, suprasternal view).

His ECG showed sinus rhythm at 70 b.p.m. and was normal. His transthoracic echocardiogram (TTE) showed a trileaflet aortic valve with no stenosis but severe regurgitation with a pressure half-time of 239.2 ms and a broad jet, reaching the left ventricular (LV) apex (see Supplementary material online, *Video S1*). There was significant holodiastolic flow reversal in the proximal descending aorta (see Supplementary material online, *Video S2*, *Figure 1*). The aorta was normal size, with no evidence of dissection. The LV was markedly dilated in diastole at 6.9 cm (3.7–5.6), and moderately in systole at 4.7 cm (2.2–4.1), with low-normal systolic function and LV ejection fraction 58.9% (≥55). The right ventricle was normal. There was no other significant valvular disease.

**Figure 2:**
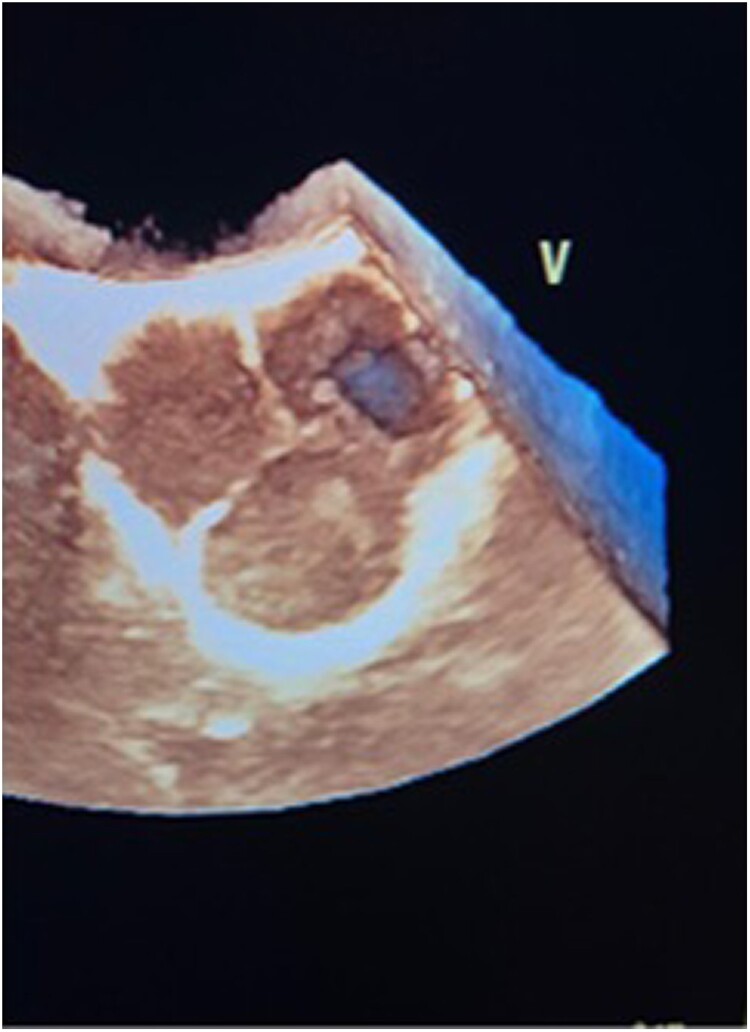
Perforation in left coronary cusp (3D TOE, aortic valve cross-section).

His transoesophageal echocardiogram (TOE) showed a trileaflet aortic valve with severe aortic regurgitation (AR) originating from the left coronary cusp (LCC) (see Supplementary material online, *Video S3*). There was a large perforation in the LCC and a prolapse of part of the LCC near the perforation (*Figure 2*). There was no evidence of dissection or vegetations.

He was discussed at a multidisciplinary meeting. In light of the history and perforation, the severe AR was felt to be secondary to trauma resulting from deceleration injury sustained in the RTA five years prior, with AR causing subsequent LV dilatation.

He underwent aortic valve replacement (AVR) with a 23 mm On-X mechanical valve prosthesis. Operative findings indicated a likely initial small tear in the LCC which had progressed, creating perforation and partial prolapse of the cusp. He made a rapid, uncomplicated recovery, was discharged on warfarin [target INR 2.5 (2–3)], and given advice about antibiotic prophylaxis against infective endocarditis per European Society of Cardiology guidelines.^3^

Nine months on, he remains well, and his ECG shows sinus rhythm at 58 b.p.m. and is normal. His echo shows a well-functioning AVR with trivial regurgitation and normal LV size, function, and wall thickness (see Supplementary material online, *Video S4*).

## Discussion

Blunt chest trauma represents over 15% of all trauma admissions to emergency departments worldwide and is the second leading cause of death in RTAs.^1^ As in our patient, BCT is commonly sustained via high-speed deceleration injury to the anterior chest. It is associated with cardiac complications, including cardiac contusion, coronary artery and/or aortic dissection, cardiac rupture, pericardial injury, arrhythmias, and, as in our patient, valvular damage and valve dysfunction.^4^ The cardiac valve most frequently injured following BCT is the aortic valve, followed by the mitral, tricuspid, and pulmonary valves.^1,5^

The incidence and prevalence of aortic valve insufficiency secondary to BCT are not defined, but it appears to be a rare but well-recognized condition. There have been isolated case reports, best reviewed in a large case series published in 2016.^6^ This found that, of the 96 included reports, car accidents accounted for 51% of cases and motorcycle accidents for 19%. Beyond the 70% of cases attributable to RTAs, other causes of AR following blunt trauma were falls (13%), falling objects (6%), sports injuries (4%), and other causes (7%).

Time to diagnosis of AR following the traumatic incident ranged from immediately to over 10 years.^6^ Early diagnoses (within 3 days) accounted for 35% of cases. A further 21% were diagnosed within 1 month and 12.5% within 3 months. In other patients, AR apparently progressed at a slower rate, with 5% diagnosed within 6 months, 4% within 1 year, 2% within 2 years, 2% within 3 years, 5% in over 5 years, and 12.5% after an unspecified duration.

The non-coronary cusp (NCC) was most frequently damaged in 55% of cases, followed by the right coronary cusp (RCC) in 36% and, least often, the LCC in 27% of cases. Mechanistically, damage most frequently affecting the NCC may be due to reduced haemodynamic stress on the RCC and LCC due to outflow into the right and left coronary arteries, respectively.^7^ The LCC may be relatively protected compared to the RCC since it is more posteriorly situated, whereas the RCC lies closer to the anterior chest wall and so may suffer higher pressure impact injury in BCT, making it more likely to tear.^7,8^

Following diagnosis, the majority (73%) of reported cases of aortic valve insufficiency after BCT were treated by AVR, with good outcomes in 89%, a mortality rate of 1%, and unspecified outcomes in 10% of patients. There was a mortality rate of five in six in patients prescribed medical therapy alone, although, importantly, these patients were too frail or declined to undergo surgery. Aortic valvuloplasty (AVP) was undertaken in 21% of patients, with good outcomes in 65%, 10% mortality rate, new-onset AR murmur in 20%, and re-operation in 5%. However, there has been a reported up-to-80% recurrence of aortic insufficiency following AVP.^9^ Hence, AVR may be preferable to AVP for treating AR following BCT.

Traumatic AR resulting from deceleration injury in our patient is reflective of other cases described in the literature, although it was relatively unusual in that damage was to the LCC. However, the timing of the presentation was much later than that commonly reported.

We present a rare complication of BCT but one that it is important for clinicians to be aware of. This case is a stark reminder that severe AR can occur even in an initially haemodynamically stable patient and remain asymptomatic, but be associated with adverse LV dilatation and remodelling which, left undiagnosed and thus untreated, can lead to irreversible ventricular dysfunction. Therefore, clinicians need to have a high index of suspicion when assessing patients with BCT, listening for new murmurs. Early diagnosis can lead to timely intervention with good clinical outcomes.

Prompt echocardiography can be invaluable in diagnosing damage to the aortic valve leaflets. Currently, there are no official guidelines recommending performing routine echocardiography in haemodynamically stable BCT patients. However, this patient’s case, and others like it, highlights the importance of routinely undertaking cardiac imaging in all patients attending the ED with significant BCT, even those who are acutely haemodynamically stable. Echocardiography is the gold standard, with TTE an invaluable screening tool, especially in the identification of valvular pathology, although diagnostic accuracy can be further enhanced by TOE if required.^10–14^ Such prompt, thorough evaluation will ensure specific diagnosis and consequently optimize treatment strategies in these patients.

## Lead author biography


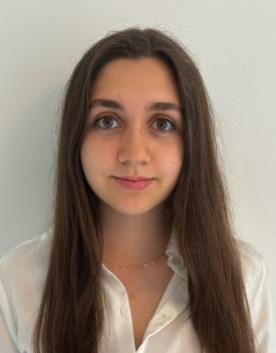


Rafaella Markides is a fourth-year medical student at the University of Cambridge.

## Supplementary Material

ytae499_Supplementary_Data

## Data Availability

The data underlying this article cannot be shared publicly for the privacy of the individual whose case is presented in this report.

## Notes

**Consent:** The authors confirm that written consent for submission and publication of this case report including the images and associated text has been obtained from the patient in line with COPE guidance.

**Funding:** None declared.
